# Development and validation of an explainable artificial intelligence model to predict in-hospital adverse events for patients with acute myocardial infarction

**DOI:** 10.1371/journal.pdig.0001812

**Published:** 2026-11-11

**Authors:** Xican Tian, Mengyuan Ji, Tao Wang, Yingliang Yang, Pixian Yuan, Yang Zhang, Congmin Zhu, Jinggang Xia, Yu Gu, Honglei Liu

**Affiliations:** 1 School of Biomedical Engineering, Capital Medical University, Beijing, China; 2 Beijing Key Laboratory of Clinical Engineering Solutions for Mental Health, Capital Medical University, Beijing, China; 3 Department of Cardiology, Xuanwu Hospital, Capital Medical University, Beijing, China; Rice University, UNITED STATES OF AMERICA

## Abstract

In-hospital adverse events after acute myocardial infarction (AMI) can occur abruptly and adversely affect short-term prognosis. We developed and externally validated explainable machine learning models to predict a composite of in-hospital atrial fibrillation (AF), ventricular fibrillation (VF), cardiac arrest (CA), or death using routinely available admission data. We retrospectively analyzed 7,496 patients from the MIMIC-IV database as the development cohort and 2,776 patients from Xuanwu Hospital as the external validation cohort. Predictors included demographics, medical history, and admission laboratory tests. Logistic regression, random forest, extreme gradient boosting (XGBoost), multilayer perceptron, and TabNet models were evaluated using five-fold cross-validation. Performance was assessed using discrimination, classification, calibration, and decision curve analysis. Latent class analysis identified three outcome clusters with distinct event-probability profiles. XGBoost achieved the best performance for the composite outcome in the development cohort (AUROC, 0.798). Predictive performance was moderate to good for AF and in-hospital death, with AUROCs of up to 0.834 and 0.870 in the development cohort and 0.803 and 0.849 in the external validation cohort, respectively. Discrimination was lower for VF and CA. SHapley Additive exPlanations highlighted neutrophil and leukocyte counts, N-terminal pro-B-type natriuretic peptide, renal function, glucose, glycated hemoglobin A1c, and prior AF as important predictors. These explainable models may support early risk stratification and help guide monitoring intensity and timely intervention in patients with AMI.

## Author summary

Acute myocardial infarction (AMI) is a life-threatening cardiovascular emergency associated with a high risk of severe in-hospital adverse events, including atrial fibrillation, ventricular fibrillation, cardiac arrest, and death. Early and accurate risk stratification using routinely available admission data remains a major clinical challenge. In this two-center retrospective study, we developed and externally validated explainable artificial intelligence models for predicting multiple in-hospital adverse events in patients with AMI. Latent class analysis was applied to identify heterogeneous phenotypic clusters of adverse events, while SHapley Additive exPlanations were used to enhance model interpretability. Our results demonstrated that tree-based models, particularly XGBoost and random forest, achieved favorable predictive performance for both composite and individual adverse events across development and external validation cohorts. Key predictors included inflammatory markers, cardiac stress indicators, renal function parameters, and metabolic biomarkers. These explainable models may support early admission-stage risk stratification, facilitate individualized monitoring strategies, and assist timely clinical intervention for hospitalized AMI patients. Overall, this study provides a clinically applicable, data-driven framework for improving inpatient risk management and optimizing resource allocation in high-risk cardiovascular care.

## Introduction

Acute myocardial infarction (AMI) is a condition caused by acute and persistent blockage of the coronary arteries, leading to myocardial ischemia and necrosis. It is one of the most common and critical cardiovascular emergencies [1]. Despite advances in reperfusion and critical care, AMI hospitalizations are frequently complicated by serious in-hospital adverse events, such as in-hospital atrial fibrillation (AF), ventricular fibrillation (VF), cardiac arrest (CA)and in-hospital death. These events typically have a rapid onset and progression, and can determine the patient’s short-term prognosis within hours to days [2–4]. Therefore, early and accurate identification of AMI patients at high risk of in-hospital adverse events is crucial. It helps in appropriately adjusting monitoring intensity, optimizing the pace of re-examinations and interventions, formulating individualized care plans, and allocating limited medical resources, with the potential to reduce sudden clinical deterioration and preventable in-hospital mortality [5].

Previous studies on predicting adverse events during AMI hospitalization have primarily used traditional models, such as logistic regression (LR) and Cox proportional hazards models [6,7]. However, such models typically rely on a limited set of manually selected predictors and assume linear effects or proportional hazards. As a result, they may not fully capture the nonlinear relationships in actual clinical data, which is often vast, high-dimensional, and missing, thereby limiting their application and further promotion in complex clinical scenarios. In recent years, machine learning models and novel deep learning methods for tabular data (such as TabNet) have increasingly been adopted because they can flexibly model nonlinear relationships and better leverage high-dimensional data with missingness. Several studies suggest that these models outperform traditional methods in discriminative ability and calibration, leading to their application in the AMI population [8]. Large registry-based studies across different countries have further shown that gradient boosting models (such as extreme gradient boosting(XGBoost) and tabular deep learning models) can exceed conventional risk scores, including Global Registry of Acute Coronary Events (GRACE) and Thrombolysis in Myocardial Infarction(TIMI), for predicting in-hospital death, with improvements in both Area Under the Receiver Operating Characteristic Curve(AUC) and risk calibration [9,10]. At the same time, some studies have used XGBoost to automatically select key features from dozens to hundreds of candidate variables to construct risk prediction models for specific adverse events, such as severe bleeding during Acute Coronary Syndrome (ACS)hospitalization. These studies demonstrate the potential of AI methods in predicting various outcomes during hospitalization [11].

However, existing studies still have notable limitations in terms of training data sources, external validation, feature engineering, model interpretability, and outcome setting [12,13]. Most models are built based on single-center or regional cohorts, with limited or no external validation, which makes it difficult to fully assess their generalizability [14,15]. Moreover, feature engineering and interpretability (e.g., clinically readable feature contributions and decision rationale) remain insufficient, affecting clinicians’ trust and adoption. Additionally, prior work primarily focuses on single outcome, such as in-hospital mortality, and lack a systematic characterization of multiple adverse events and their co-occurrence during hospitalization [16].

Against this backdrop, we conducted a two-center retrospective study in hospitalized AMI patients. We collected structured variables early after admission, including demographic characteristics, biochemical indicators, and medical history, from a tertiary hospital in China (Xuanwu Hospital), which was used as the external validation cohort. In addition, we extracted eligible AMI admissions from the MIMIC-IV (version 2.2) database, which served as the development cohort for model construction. Based on the application scenario of early in-hospital risk stratification, this study prioritized the selection of representative in-hospital adverse events that are clinically significant, with clear endpoint definitions and relatively stable records in both cohorts. These events are ranked by severity as follows: AF, VF, CA, and in-hospital death. This study aimed to construct individualized risk prediction models based on these events. Machine learning and deep learning models, including LR, random forest (RF), XGBoost, multilayer perceptron (MLP) and TabNet, were used as the core models. Decision curve analysis (DCA) and calibration evaluation were further adopted to validate clinical applicability and predictive robustness. Meanwhile, we employed SHapley Additive exPlanations (SHAP) to explore model interpretability, and applied latent class analysis (LCA) to characterize distinct patterns of adverse-event co-occurrence. Unlike previous studies that primarily focused on single endpoints, this research simultaneously models and evaluates multiple clinically relevant adverse events, aiming to more comprehensively characterize the risk spectrum during AMI hospitalization.

## Method

### Ethics statement

MIMIC-IV is a publicly available, deidentified critical care database jointly curated by the Massachusetts Institute of Technology (Cambridge, MA, USA) and Beth Israel Deaconess Medical Center (Boston, MA, USA). Use of MIMIC-IV has been approved by the institutional review boards of both institutions, and the requirement for individual informed consent was waived because all data are deidentified. One author (XT) completed the required training in human research participant protection and obtained certification (record ID 69083329), enabling access to MIMIC-IV.

The study protocol for the Xuanwu Hospital cohort was approved by the Ethics Committee of Xuanwu Hospital (approval No. 2022–129) and conducted in accordance with the Declaration of Helsinki. All enrolled patients in the Xuanwu cohort provided written informed consent.

### Dataset

This retrospective study included two cohorts: (1) 2,993 consecutive patients diagnosed with AMI and admitted to Xuanwu Hospital, Capital Medical University between January 2017 and December 2022; (2) 16,288 AMI admissions identified from MIMIC-IV database [17].

We defined index time as hospital admission. For both cohorts, all laboratory predictors were extracted as the first available test results during hospitalization (earliest post‑admission value). Demographic variables were time‑independent. All predictors were collected prior to outcomes to prevent label leakage. Baseline demographics included sex, age, height, weight, and body mass index (BMI). Medical history variables included hypertension, diabetes, hyperlipidemia, prior myocardial infarction, previous percutaneous coronary intervention, previous coronary artery bypass grafting (CABG), previous stroke, prior AF, and smoking history, along with types of AMI. All laboratory values were the earliest recorded blood test results after admission in both MIMIC‑IV and Xuanwu Hospital. Blood test results included random glucose, Glycated Hemoglobin A1c (HbA1c), creatinine, creatinine clearance, total cholesterol, low-density lipoprotein cholesterol (LDL-C), high-density lipoprotein cholesterol (HDL-C), triglycerides, uric acid, N-terminal pro–B-type natriuretic peptide (NT-proBNP), white blood cell count, neutrophils, lymphocytes, and monocytes.

The prediction tasks focused on four in-hospital adverse events: AF, VF, CA, and in-hospital death. The four in-hospital adverse events were identified using International Classification of Diseases (ICD) diagnosis codes from final discharge diagnoses in both cohorts. To ensure independent observations and focus on incident in-hospital adverse events during the index AMI admission, only the first hospitalization for AMI was included per patient. Subsequent or multiple admissions were defined as duplicate admissions and excluded. We restricted analyses to events occurring during the index admission. After excluding patients with missing data in key variables and removing duplicate or multiple admissions, 7,496 unique AMI admissions from MIMIC-IV were used as the development cohort, and 2,776 patients from Xuanwu Hospital comprised the external validation cohort (Fig 1). Baseline laboratory values were checked for distribution, units and outliers. We unified measurement indicators across two cohorts, with consistent unit specifications detailed in S1 Table. Moderate missing data existed in features, around 25% variables with excessive missingness were discarded. Median imputation was adopted for continuous variables and the most frequent category was used for missing categorical variables in the development cohort; the corresponding imputation rules derived from the development cohort were subsequently applied to the external validation cohort. Valid clinical readings were retained uniformly.

**Fig 1:**
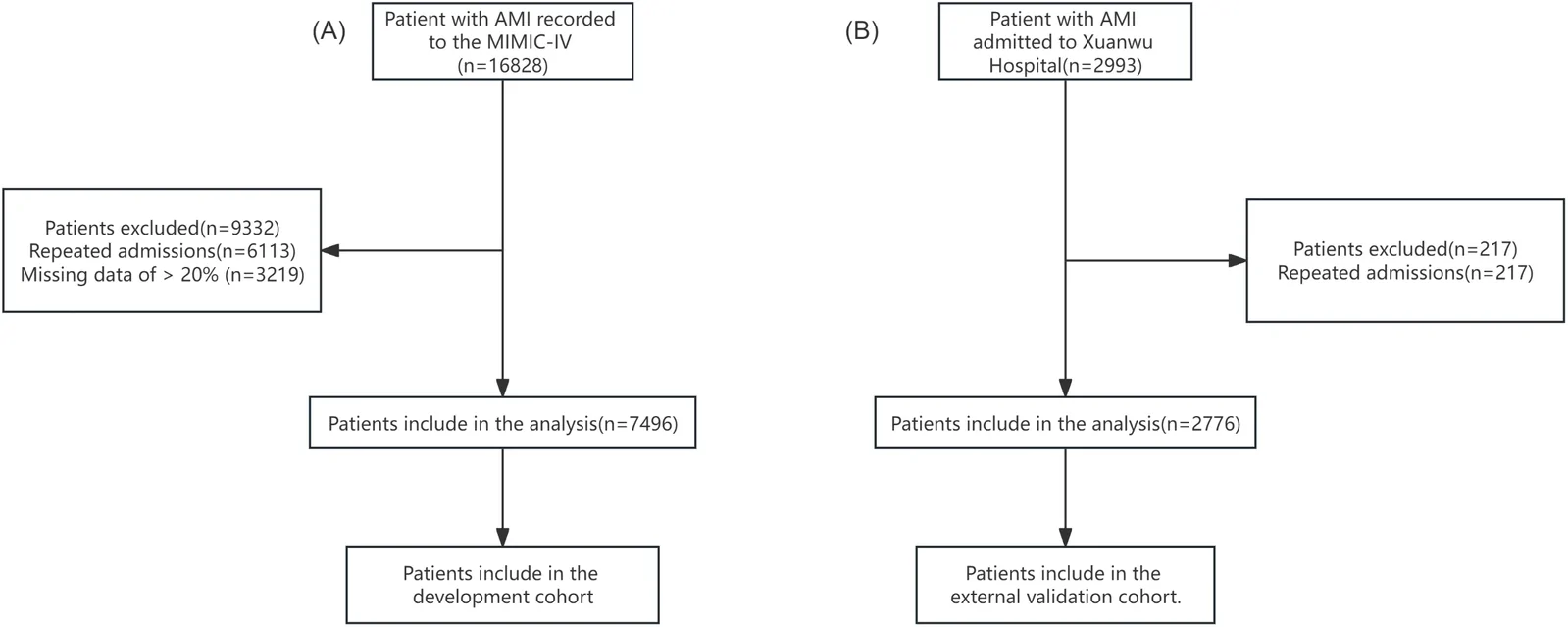
Inclusion and exclusion criteria for the (A)development cohort and (B) external validation cohort.

### Model construction and comparison

Five algorithms for structured clinical data were evaluated: LR, RF, XGBoost, MLP and TabNet. All models were developed, tuned on the MIMIC‑IV cohort (development cohort) using 5‑fold stratified cross‑validation. In each fold, approximately 80% of samples were used for training and 20% for validation while preserving the original outcome distribution. Prediction probabilities from all validation folds were aggregated for overall model evaluation, and a fixed classification threshold of 0.5 was applied across all models to ensure consistency and comparability. The Xuanwu Hospital cohort was used solely as the external validation cohort. Discrimination was evaluated using AUROC as the primary metric, complemented by AUPRC, precision, and F1-score to reflect performance under class imbalance. Model predictions were explained using SHAP to quantify feature-level contributions. Calibration was assessed using calibration curves, Brier score, calibration slope, and calibration intercept, while clinical utility was evaluated using DCA. We also calculated sensitivity, specificity, positive predictive value (PPV), negative predictive value (NPV), and quantified the alert rate per 100 patients for decision-oriented clinical evaluation. To ensure reproducibility, the final optimized hyperparameters for all models and the complete software environment are provided in S2 Table. To quantify statistical uncertainty, 95% confidence intervals (95% CIs) for all performance metrics were estimated using nonparametric bootstrap resampling with 2,000 iterations.

In addition, LCA was conducted on the four binary outcomes using a Bernoulli mixture model (K = 3) fitted by the EM algorithm; heatmaps were used to visualize class-specific event probabilities and characterize outcome-pattern heterogeneity across subgroups. SHAP dependence plots were further adopted to visualize the varying influence of core predictors across different value ranges.

## Results

### Patient characteristics

After applying the eligibility criteria, 7,496 AMI admissions in MIMIC-IV constituted the development cohort, and 2,776 AMI patients from Xuanwu Hospital comprised the external validation cohort (Fig 1). Baseline demographics, comorbidities, and admission-stage laboratory and other key clinical variables are summarized in Table 1. The distributions of major predictors stratified by each adverse event in the Xuanwu cohort are provided in S1 Table.

**Table 1:** Baseline characteristics of patients in the MIMIC-IV (development) and Xuanwu Hospital (external validation) cohorts.

Variables	MIMIC-IV (n=7496)	Xuanwu (n=2776)
**Demographic Characteristics**, **Median (IQR)**		
Sex, Male, n (%)	2733 (36.5%)	2154 (77.6%)
Age	69.00 (60.00-78.00)	63.00 (55.00-71.00)
Heigh,cm	170.00(168.00-175.00)	170.00 (163.00-173.00)
Weight,kg	81.50 (75.00-89.00)	72.00 (65.00-80.00)
BMI	28.04 (26.08-30.19)	25.35 (23.38-27.68)
**Medical History,n(%)**		
Hypertension	6198 (82.7%)	1667 (60.1%)
Diabetes	2391 (31.9%)	1014 (36.5%)
Hyperlipidemia	5162 (68.9%)	442 (15.9%)
Prior Myocardial Infarction	0 (0.0%)	318 (11.5%)
Previous PCI	1179 (15.7%)	346 (12.5%)
Previous CABG	900 (12.0%)	40 (1.4%)
Previous Stroke	1501 (20.0%)	371 (13.4%)
Previous Atrial Fibrillation	918 (12.2%)	110 (4.0%)
Smoking History	816 (10.9%)	1578 (56.8%)
**Blood Test Results, Median(IQR)**		
Glucose, mmol/L	9.50 (7.39-13.44)	5.92 (5.02-8.11)
HbA1c,%	5.90 (5.90-5.90)	6.00 (5.60-7.10)
Creatinine, µmol/L	97.24 (70.72-141.44)	74.00 (63.00-87.00)
Creatinine Clearance, mL/min	78.29 (61.05-96.93)	83.15 (62.32-106.33)
Total Cholesterol, mmol/L	4.47 (3.83-5.25)	4.06 (3.45-4.78)
LDL-C, mmol/L	3.40 (3.00-3.70)	2.52 (2.03-3.12)
HDL-C, mmol/L	4.42 (3.44-6.26)	0.98 (0.83-1.16)
Triglycerides, mmol/L	1.14 (1.03-1.25)	1.48 (1.07-2.13)
Uric Acid, µmol/L	374.72(374.72-374.72)	346.00 (282.75-417.00)
NT-ProBNP, ng/L	186.00(137.00-316.00)	514.00(171.00-1492.25)
White Blood Cell Count,×10^9/L	9.50 (7.20-12.70)	9.02 (7.30-11.25)
Neutrophils,×10^9/L	8.84 (8.75-8.95)	6.57 (4.77-8.93)
Lymphocytes,×10^9/L	1.40 (1.38-1.40)	1.60 (1.17-2.12)
Monocytes,×10^9/L	0.60 (0.35-0.95)	0.46 (0.35-0.60)
**Types of AMI, n(%)**		
STEMI	3023 (40.3%)	1675 (60.3%)

Continuous variables are presented as **median (interquartile range) [median (IQR)]**, and categorical variables are expressed as **number (percentage) [n (%)]**.

Abbreviations: **BMI,** body mass index; **PCI**,Percutaneous Coronary Intervention;**CABG**, coronary artery bypass grafting; **HbA1c,** Glycated Hemoglobin A1c; **LDL-C**, low-density lipoprotein cholesterol; **HDL-C**, high-density lipoprotein cholesterol; **NT-proBNP**, N-terminal pro–B-type natriuretic peptide; **STEMI**, ST-segment elevation myocardial infarction.

Overall, the two cohorts showed substantial differences in demographic composition, burden of cardiovascular risk factors and comorbidities, and key laboratory profiles, reflecting distinct case-mix and practice patterns between the two datasets.

### Outcome phenotypes identified by LCA

LCA of the four binary outcomes supported a three-class solution (K = 3) in both cohorts, as indicated by the minimum Bayesian Information Criterion (MIMIC-IV cohort BIC = 17,473.70; Xuanwu cohort BIC = 3,345.37). Class-specific event probabilities are visualized as a heatmap in Fig 2, highlighting distinct outcome-pattern phenotypes and subgroup-level risk heterogeneity.

**Fig 2:**
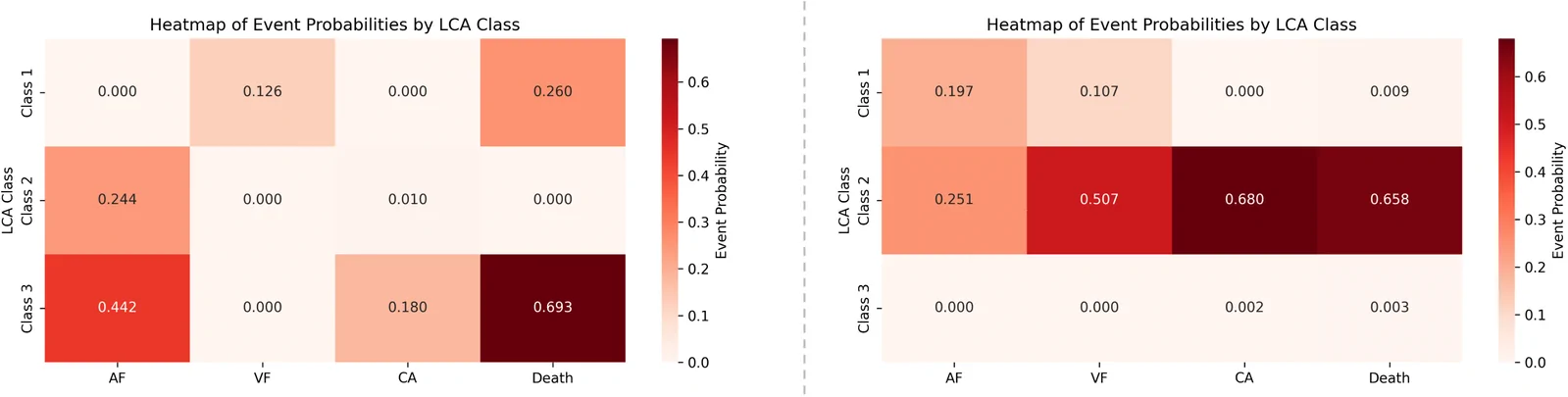
Heatmaps of adverse-event probabilities across LCA-derived latent outcome classes in the MIMIC-IV and Xuanwu cohorts. (A) MIMIC-IV cohort. **(B)** Xuanwu cohort.

LCA suggested that CA frequently co-occurred with other adverse events, whereas VF appeared comparatively independent of AF and CA. AF occurring in isolation rarely coincided with in-hospital death, while VF and CA showed a closer association with in-hospital death, implying that AF may carry lower immediate severity than VF or CA. This observation is consistent with the conventional framing of major adverse cardiac events (MACE), which typically includes VF, CA, and death, but not AF. In addition, the LCA was not used for risk stratification, model training, or subgroup-specific prediction. In the MIMIC-IV cohort, Class 1, Class 2, and Class 3 accounted for 41.2%, 33.5%, and 25.3% of patients, respectively; in the Xuanwu cohort, the distribution was 38.7%, 35.1%, and 26.2%. The high-risk Class 2 in Xuanwu was characterized by markedly elevated probabilities of VF (50.7%), CA (68.0%), and in-hospital death (65.8%), confirming its clinical relevance as a severe adverse event phenotype.

### Model performance

Model performance is presented in two parts: (1) prediction of the composite outcome—occurrence of any in-hospital adverse event (Table 2 and Fig 3); and (2) prediction of each component event separately—AF, VF, CA and in-hospital death (Table 3, S3 Table, Fig 4, S1 and S2 Figs, S4 Table, S4-S6 Figs).

**Table 2:** Performance of RF, XGBoost, MLP, TabNet and LR for predicting any in-hospital adverse event in the MIMIC-IV (development) and Xuanwu (external test) cohorts.

(A) MIMIC-IV development cohort					
MIMIC(development cohort)					
Models	RF	XGBoost	MLP	TabNet	LR
**AUROC(95% CI)**	0.783(0.772 ~ 0.794)	0.798(0.788 ~ 0.809)	0.755(0.742 ~ 0.766)	0.756(0.744 ~ 0.767)	0.745(0.733 ~ 0.756)
**AUPRC(95% CI)**	0.659(0.640 ~ 0.679)	0.683(0.664 ~ 0.702)	0.617(0.597 ~ 0.638)	0.622(0.602 ~ 0.642)	0.600(0.580 ~ 0.621)
**Accuracy(95% CI)**	0.718(0.708 ~ 0.729)	0.730(0.720 ~ 0.740)	0.721(0.710 ~ 0.731)	0.718(0.708 ~ 0.728)	0.697(0.686 ~ 0.707)
**F1-score(95% CI)**	0.629(0.614 ~ 0.644)	0.646(0.631 ~ 0.660)	0.548(0.529 ~ 0.565)	0.568(0.550 ~ 0.585)	0.604(0.590 ~ 0.619)
**Sensitivity(95% CI)**	0.671(0.653 ~ 0.690)	0.694(0.676 ~ 0.712)	0.476(0.456 ~ 0.495)	0.522(0.503 ~ 0.541)	0.653(0.635 ~ 0.671)
**Specificity(95% CI)**	0.744(0.732 ~ 0.756)	0.749(0.737 ~ 0.762)	0.855(0.846 ~ 0.865)	0.826(0.815 ~ 0.836)	0.721(0.708 ~ 0.734)
**PPV(95% CI)**	0.591(0.574 ~ 0.608)	0.604(0.588 ~ 0.621)	0.644(0.624 ~ 0.666)	0.622(0.602 ~ 0.643)	0.563(0.546 ~ 0.580)
**NPV(95% CI)**	0.805(0.794 ~ 0.816)	0.817(0.805 ~ 0.828)	0.748(0.737 ~ 0.759)	0.758(0.747 ~ 0.770)	0.791(0.778 ~ 0.802)
**Alert Rateper 100 patients(95% CI)**	40.3(39.3 ~ 41.4)	40.8(39.7 ~ 42.0)	26.2(25.2 ~ 27.2)	29.8(28.8 ~ 30.8)	41.2(40.1 ~ 42.3)
**Brier Score(95% CI)**	0.187(0.183 ~ 0.190)	0.179(0.175 ~ 0.184)	0.188(0.183 ~ 0.193)	0.191(0.185 ~ 0.196)	0.205(0.202 ~ 0.209)
**Calibration** **Slope(95% CI)**	1.254(1.187 ~ 1.325)	0.952(0.906 ~ 1.002)	0.861(0.797 ~ 0.922)	0.656(0.615 ~ 0.698)	0.936(0.852 ~ 1.018)
**Calibration Intercept(95% CI)**	-0.456(-0.508 ~ -0.404)	-0.480(-0.533 ~ -0.426)	-0.089(-0.147 ~ -0.025)	-0.154(-0.212 ~ -0.095)	-0.598(-0.648 ~ -0.547)
**(B) Xuanwu external test cohort**					
**Xuanwu(external validation cohort)**					
**Models**	**RF**	**XGBoost**	**MLP**	**TabNet**	**LR**
**AUROC(95% CI)**	0.714(0.681 ~ 0.747)	0.711(0.677 ~ 0.743)	0.742(0.711 ~ 0.773)	0.633(0.598 ~ 0.665)	0.765(0.736 ~ 0.793)
**AUPRC(95% CI)**	0.308(0.257 ~ 0.361)	0.307(0.254 ~ 0.359)	0.329(0.276 ~ 0.382)	0.168(0.142 ~ 0.197)	0.362(0.306 ~ 0.419)
**Accuracy(95% CI)**	0.783(0.768 ~ 0.798)	0.784(0.769 ~ 0.800)	0.893(0.882 ~ 0.904)	0.810(0.795 ~ 0.824)	0.874(0.862 ~ 0.887)
**F1-score(95% CI)**	0.339(0.300 ~ 0.378)	0.346(0.306 ~ 0.383)	0.242(0.188 ~ 0.297)	0.233(0.190 ~ 0.275)	0.400(0.345 ~ 0.451)
**Sensitivity(95% CI)**	0.508(0.452 ~ 0.560)	0.517(0.462 ~ 0.574)	0.154(0.116 ~ 0.195)	0.262(0.214 ~ 0.311)	0.384(0.330 ~ 0.439)
**Specificity(95% CI)**	0.817(0.802 ~ 0.832)	0.817(0.801 ~ 0.832)	0.985(0.979 ~ 0.989)	0.877(0.864 ~ 0.890)	0.934(0.924 ~ 0.944)
**PPV(95% CI)**	0.255(0.221 ~ 0.292)	0.259(0.224 ~ 0.293)	0.552(0.451 ~ 0.656)	0.208(0.167 ~ 0.250)	0.419(0.359 ~ 0.474)
**NPV(95% CI)**	0.931(0.920 ~ 0.942)	0.932(0.922 ~ 0.942)	0.904(0.893 ~ 0.915)	0.906(0.894 ~ 0.917)	0.925(0.915 ~ 0.935)
**Alert Rateper 100 patients(95% CI)**	21.8(20.4 ~ 23.5)	22.0(20.5 ~ 23.5)	3.1(2.4 ~ 3.7)	13.8(12.6 ~ 15.1)	10.1(8.9 ~ 11.2)
**Brier Score(95% CI)**	0.171(0.167 ~ 0.175)	0.160(0.154 ~ 0.166)	0.089(0.082 ~ 0.096)	0.144(0.134 ~ 0.153)	0.126(0.121 ~ 0.131)
**Calibration** **Slope(95% CI)**	1.468(1.215 ~ 1.724)	0.834(0.704 ~ 0.973)	0.963(0.746 ~ 1.168)	0.179(0.101 ~ 0.262)	1.179(0.591 ~ 1.634)
**Calibration Intercept(95% CI)**	-1.652(-1.780 ~ -1.534)	-1.740(-1.860 ~ -1.627)	-0.514(-0.848 ~ -0.210)	-1.766(-1.961 ~ -1.596)	-1.319(-1.683 ~ -1.054)

**Table 3:** Performance of RF, XGBoost, MLP, TabNet and LR for predicting four in-hospital adverse events (AF, VF, CA, and in-hospital death) in the MIMIC-IV cohort.

MIMIC(development cohort)						
AE	Models	RF	XGBoost	MLP	TabNet	LR
**AF**	**AUROC(95% CI)**	0.828(0.819 ~ 0.838)	0.834(0.825 ~ 0.844)	0.748(0.736 ~ 0.761)	0.761(0.749 ~ 0.774)	0.741(0.728 ~ 0.754)
	**AUPRC(95% CI)**	0.595(0.572 ~ 0.619)	0.614(0.590 ~ 0.636)	0.493(0.468 ~ 0.519)	0.506(0.481 ~ 0.531)	0.473(0.448 ~ 0.497)
	**F1-score(95% CI)**	0.579(0.562 ~ 0.596)	0.588(0.570 ~ 0.605)	0.390(0.368 ~ 0.414)	0.427(0.404 ~ 0.450)	0.508(0.489 ~ 0.525)
	**Sensitivity(95% CI)**	0.729(0.708 ~ 0.750)	0.744(0.724 ~ 0.765)	0.291(0.271 ~ 0.314)	0.348(0.325 ~ 0.371)	0.653(0.631 ~ 0.675)
**VF**	**AUROC(95% CI)**	0.832(0.802 ~ 0.859)	0.840(0.809 ~ 0.868)	0.590(0.536 ~ 0.644)	0.651(0.594 ~ 0.703)	0.740(0.696 ~ 0.780)
	**AUPRC(95% CI)**	0.063(0.047 ~ 0.083)	0.074(0.053 ~ 0.102)	0.025(0.018 ~ 0.034)	0.037(0.025 ~ 0.050)	0.051(0.036 ~ 0.072)
	**F1-score(95% CI)**	0.105(0.083 ~ 0.126)	0.112(0.090 ~ 0.135)	0.000(0.000 ~ 0.000)	0.000(0.000 ~ 0.000)	0.064(0.050 ~ 0.078)
	**Sensitivity(95% CI)**	0.643(0.555 ~ 0.733)	0.701(0.618 ~ 0.782)	0.000(0.000 ~ 0.000)	0.000(0.000 ~ 0.000)	0.627(0.538 ~ 0.711)
**CA**	**AUROC(95% CI)**	0.765(0.735 ~ 0.793)	0.767(0.737 ~ 0.794)	0.562(0.529 ~ 0.599)	0.661(0.626 ~ 0.694)	0.728(0.697 ~ 0.758)
	**AUPRC(95% CI)**	0.106(0.084 ~ 0.131)	0.106(0.084 ~ 0.133)	0.054(0.040 ~ 0.073)	0.064(0.052 ~ 0.077)	0.081(0.067 ~ 0.097)
	**F1-score(95% CI)**	0.183(0.143 ~ 0.222)	0.155(0.133 ~ 0.179)	0.016(0.000 ~ 0.040)	0.000(0.000 ~ 0.000)	0.134(0.115 ~ 0.155)
	**Sensitivity(95% CI)**	0.260(0.206 ~ 0.313)	0.549(0.489 ~ 0.611)	0.008(0.000 ~ 0.021)	0.000(0.000 ~ 0.000)	0.608(0.550 ~ 0.667)
**Death**	**AUROC(95% CI)**	0.853(0.841 ~ 0.863)	0.870(0.860 ~ 0.880)	0.836(0.824 ~ 0.847)	0.823(0.810 ~ 0.835)	0.827(0.814 ~ 0.840)
	**AUPRC(95% CI)**	0.477(0.444 ~ 0.509)	0.507(0.475 ~ 0.541)	0.447(0.416 ~ 0.481)	0.409(0.379 ~ 0.440)	0.408(0.379 ~ 0.439)
	**F1-score(95% CI)**	0.498(0.475 ~ 0.519)	0.505(0.484 ~ 0.526)	0.316(0.285 ~ 0.347)	0.347(0.317 ~ 0.379)	0.465(0.443 ~ 0.485)
	**Sensitivity(95% CI)**	0.688(0.658 ~ 0.716)	0.764(0.738 ~ 0.789)	0.214(0.190 ~ 0.239)	0.267(0.241 ~ 0.295)	0.733(0.705 ~ 0.760)

**Fig 3:**
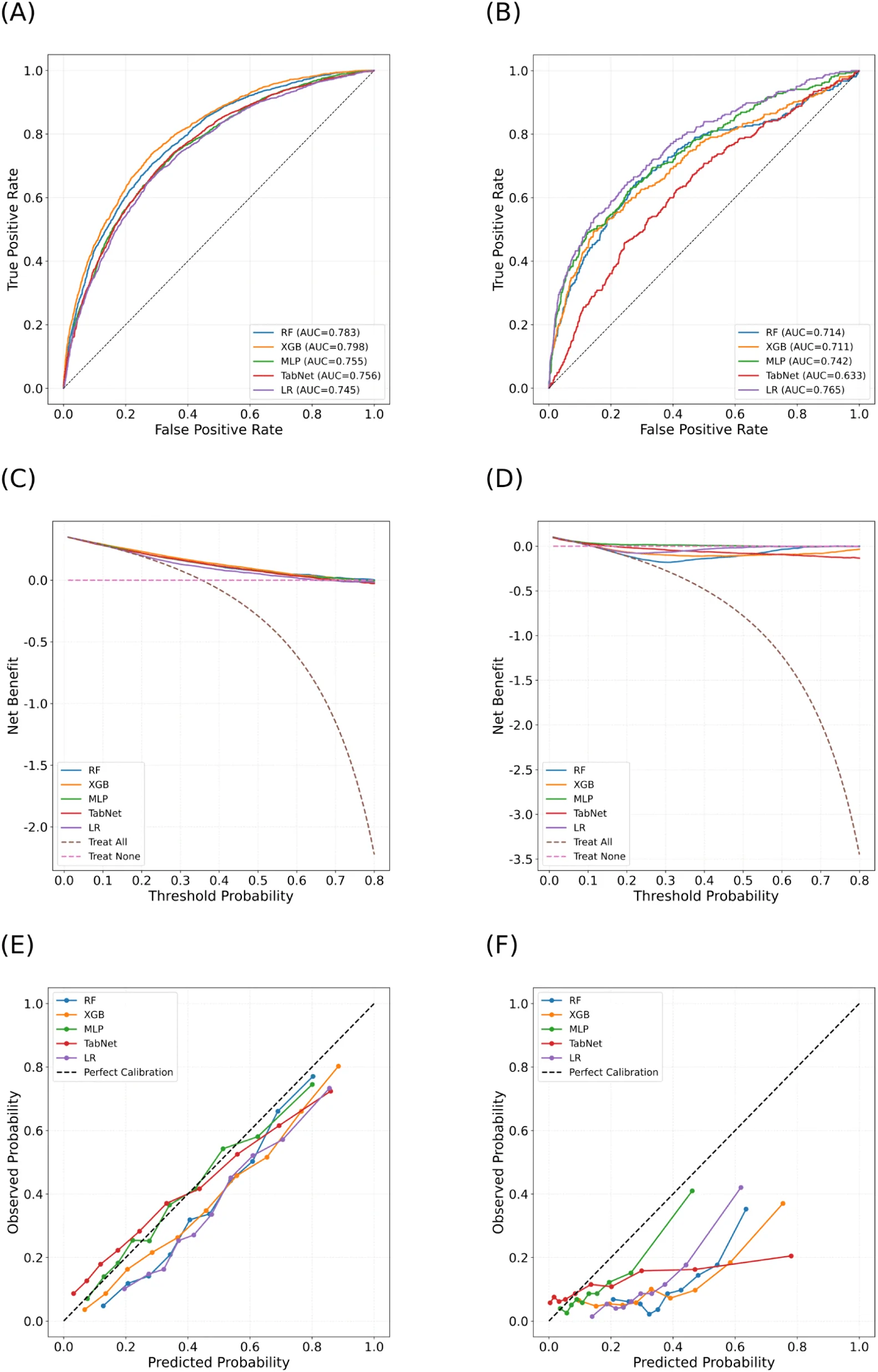
ROC curves for predicting any in-hospital adverse event in the MIMIC-IV and Xuanwu cohorts. **(A)** MIMIC-IV cohort. **(B)** Xuanwu cohort. DCA curves for predicting any in-hospital adverse event in the MIMIC-IV and Xuanwu cohorts. **(C)** MIMIC-IV cohort. **(D)** Xuanwu cohort. Calibration curves for predicting any in-hospital adverse event in the MIMIC-IV and Xuanwu cohorts. **(E)** MIMIC-IV cohort. **(F)** Xuanwu cohort.

**Fig 4:**
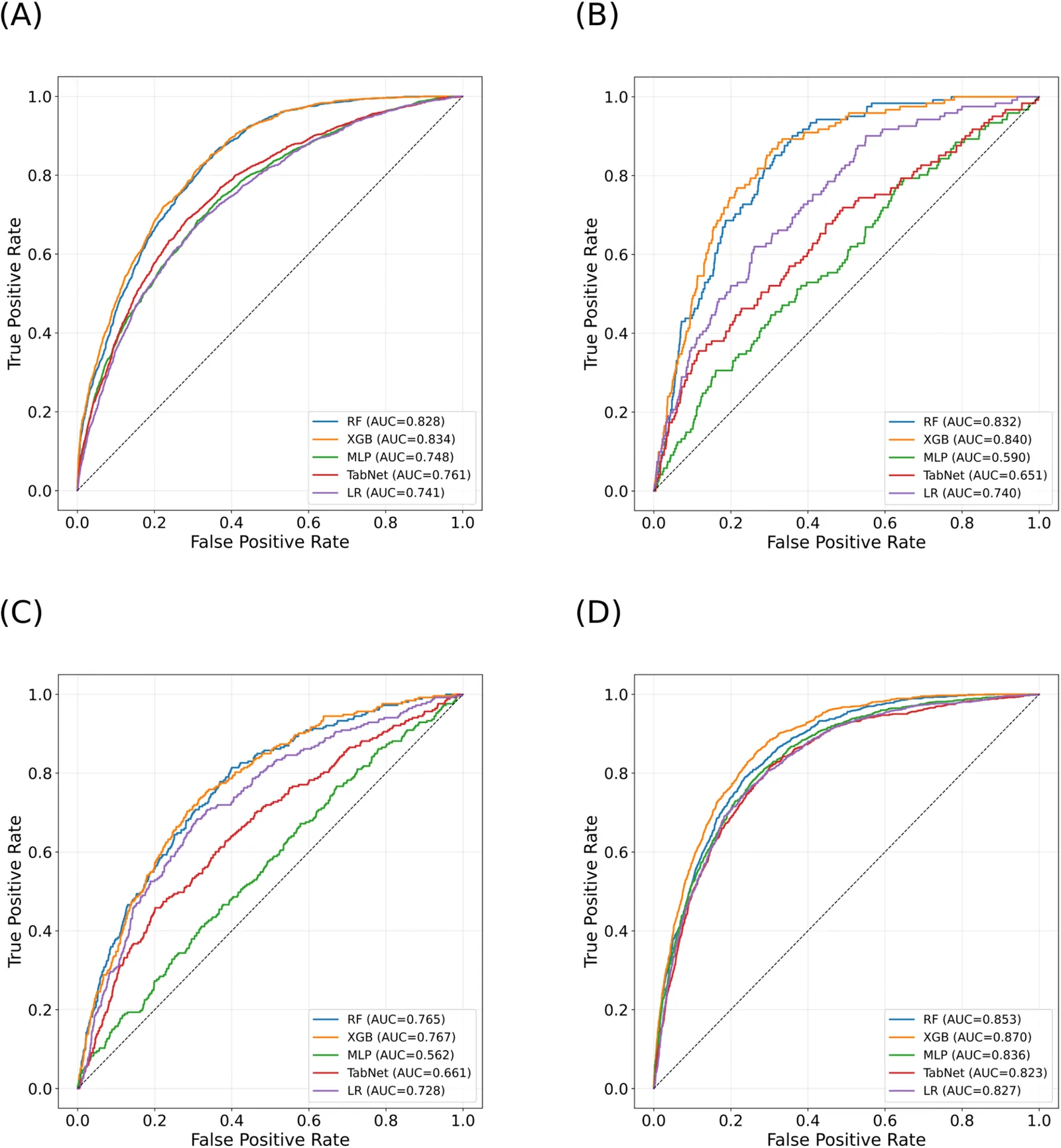
ROC curves for predicting four in-hospital adverse events in the MIMIC-IV cohort. (A) AF. **(B)** VF. **(C)** CA. **(D)** In-hospital death.

For the composite outcome, XGBoost achieved the best discrimination in the MIMIC-IV development cohort, with an AUROC of 0.798 (0.788 - 0.809). RF performed comparably, with an AUROC of 0.783 (0.772 - 0.794) (Table 2A and Fig 3). In the external Xuanwu cohort, LR yielded the highest AUROC (0.765, 0.736 - 0.793) (Table 2B and Fig 3). Overall, all metrics declined in development cohort relative to the external validation cohort results.

For the event-specific models in the MIMIC-IV development cohort, all four outcomes demonstrated moderate-to-high discrimination. Based on AUROC, the best-performing models were XGBoost for AF (0.834, 0.825 - 0.844), XGBoost for VF (0.840, 0.809 - 0.868), XGBoost for CA (0.767, 0.737 - 0.794), and XGBoost for in-hospital death (0.870, 0.860 - 0.880) (Table 3, S3 Table, Fig 4). Beyond discriminative performance, clinical decision-relevant indicators were summarized to reflect practical utility. For illustration, in the prediction of VF, the XGBoost model demonstrated a moderate specificity (0.822) and a high NPV (0.994), with an alert rate of 18.6 per 100 patients, indicating good rule-out performance with an acceptable clinical alert burden. Full data across all outcomes and algorithms are available in S3 Table. In the external validation cohort, performance decreased across outcomes. The highest AUROCs were achieved by LR for AF (0.803, 0.764 - 0.839), MLP for VF (0.566, 0.512 - 0.619), RF for CA (0.747, 0.679 - 0.812), and RF for in-hospital death (0.849 0.802 - 0.891) (S4 Table, S4 Fig). Beyond AUROC, the best-performing external model for in-hospital death prediction (RF) also demonstrated excellent clinical utility, with very high specificity (0.991) and NPV (0.975), while generating only 1.5 alert rate per 100 patients. These findings suggest that the model may be useful for identifying patients at low risk of in-hospital death while minimizing unnecessary clinical alerts.

In summary, models yielded clinically promising predictive performance for both composite adverse outcome and individual endpoint prediction. Tree-based algorithms including RF and XGBoost showed steady superiority over deep learning models MLP and TabNet in discriminative ability and generalization robustness. Notably, prediction efficacy was generally lower in the external validation cohort than in the development cohort.

### Model interpretability

To further interpret the prediction models, we performed SHAP analyses to quantify the contribution of individual features to model outputs. Fig 5 shows SHAP summary plots for the best-performing models trained in the MIMIC-IV cohort, including the composite outcome evaluated in both cohorts, and event-specific outcomes evaluated in MIMIC-IV cohort. SHAP summary plots for the corresponding event-specific models in the Xuanwu cohort are provided in S7 Fig. To facilitate interpretation of the most influential predictors, SHAP dependence plots for the top six features in the best-performing MIMIC-IV models were included, illustrating how each variable’s value range influences the model’s predicted risk (S3 Fig).

**Fig 5:**
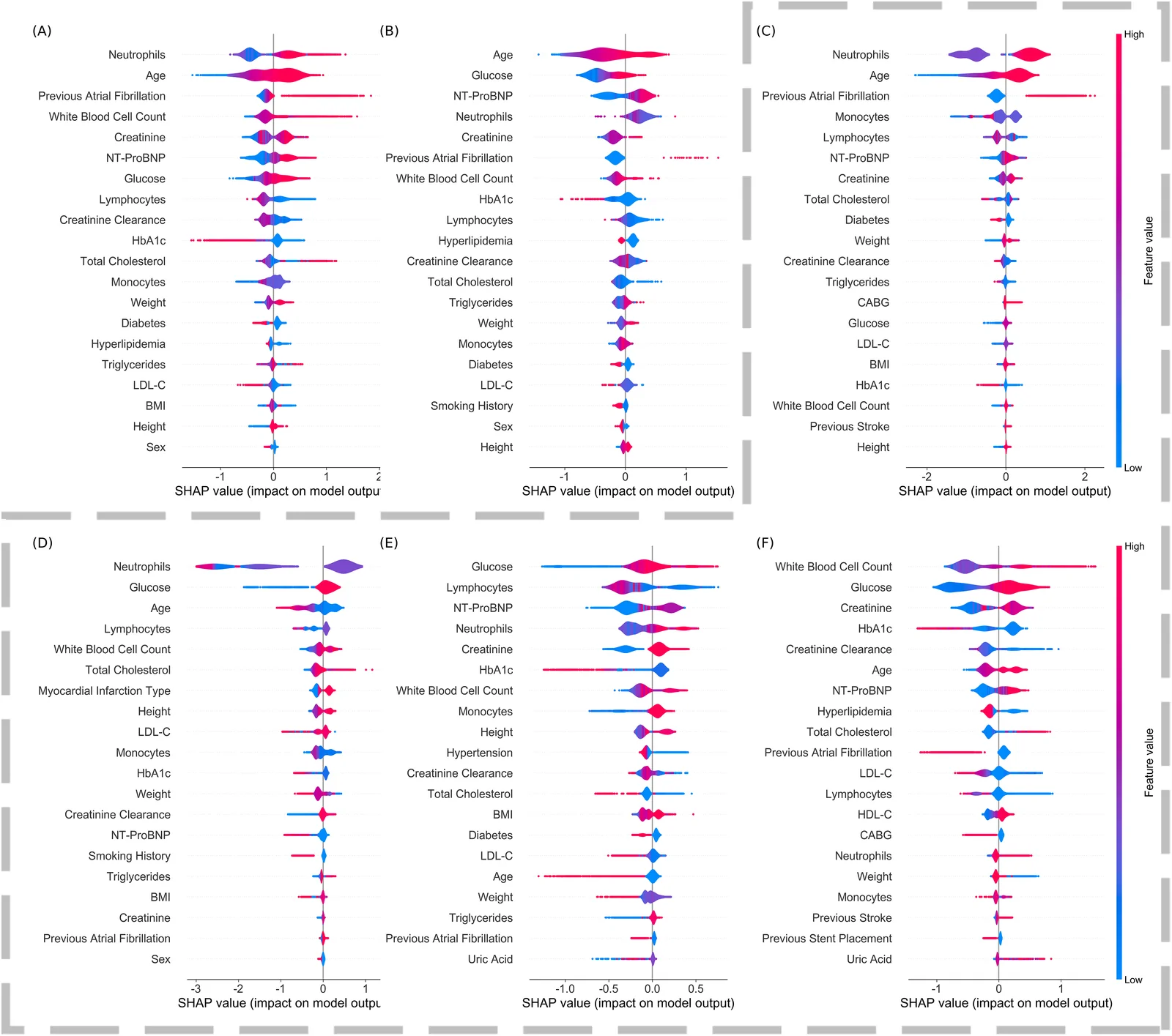
SHAP summary plots for the best-performing models across cohorts and outcomes. **(A–B)** Any in-hospital adverse event in the MIMIC-IV and Xuanwu cohorts, respectively. **(C–F)** AF, VF, CA, and in-hospital death in the MIMIC-IV cohort, respectively.

For the composite outcome, neutrophil count and age were identified as important predictors in both cohorts (Fig 5A–B). Variables related to renal function and cardiac stress, particularly creatinine and NT-proBNP, also contributed substantially to model predictions across cohorts. Metabolic indicators, including glucose, HbA1c, and lipid-related variables, were likewise associated with predicted in-hospital adverse event risk. Examination of SHAP dependence plots suggested that higher age, neutrophil count, and creatinine levels were generally associated with increased predicted risk, while previous atrial fibrillation also contributed positively to adverse event prediction.

Across event-specific outcomes in the Xuanwu cohort, the SHAP summary plots revealed patterns broadly consistent with those observed in the MIMIC-IV cohort, with inflammation-, metabolic-, and organ dysfunction-related variables consistently ranking among the most important predictors, alongside several outcome-specific differences (S7 FigA–D). For AF in the Xuanwu cohort (S7 FigA), inflammatory markers (e.g., neutrophils and monocytes), age, and prior AF history ranked among the most influential features, showing patterns generally similar to those observed in the MIMIC-IV cohort (Fig 5C). SHAP dependence plots (S3 FigB) further demonstrated generally positive associations of age and neutrophil levels with model-predicted risk contribution, while prior AF history showed a marked positive contribution to model output. For VF (S7 FigB), key predictors included neutrophils, glucose, lymphocytes, monocytes. Similar to the MIMIC-IV cohort (Fig 5D), inflammatory and metabolic variables remained prominent contributors to model predictions. For CA (S7 FigC), glucose, HbA1c, creatinine, NT-proBNP, and lymphocytes ranked highly in the model. These findings were generally consistent with the MIMIC-IV cohort (Fig 5E), where metabolic, inflammatory, and renal function-related variables also showed strong associations with model-predicted CA risk. For in-hospital death (S7 FigD), glucose, white blood cell count, and creatinine were among the top-ranked predictors, with inflammatory, renal, and metabolic variables also contributing substantially to model output. This pattern broadly resembled the findings observed in the MIMIC-IV cohort (Fig 5F). The overall consistency of key predictors across cohorts indicates satisfactory model robustness within the present study. Outcome-specific differences in feature importance, meanwhile, reflect the distinct pathophysiological mechanisms underlying each adverse event, further supporting the clinical validity of the identified predictors.

## Discussion

This study developed individualized models to predict common in-hospital adverse events after AMI, including AF, VF, CA, and in-hospital death, using routinely available structured clinical data. Using cohorts from MIMIC-IV and Xuanwu Hospital, we evaluated RF, XGBoost, MLP, and TabNet. Overall, all models showed meaningful discrimination across outcomes. This model is intended for admission-based risk screening in AMI patients. A high-risk score will trigger enhanced cardiac monitoring, intensified clinical observation and targeted preventive interventions, while low-risk individuals can follow standard inpatient care protocols. Model interpretation with SHAP identified key predictors and their directions of effect, improving transparency and aligning model outputs with clinical reasoning; the top-ranked features were broadly consistent with prior AMI prognostic evidence and clinical experience.

Across cohorts, performance was higher in the MIMIC-IV development cohort than in the Xuanwu external cohort, but the decline was modest, indicating reasonable generalizability. The reduction in external performance may reflect differences in data distributions, and practice patterns between a large, heterogeneous multi-source database (MIMIC-IV) and a single-center cohort (Xuanwu).

In model comparisons, tree-based methods showed better discrimination and robustness across both cohorts. Compared with LR, MLP and TabNet, RF and XGBoost achieved higher AUROC, AUPRC, Sensitivity, and F1 in most tasks, and their performance was less affected by class imbalance and between-cohort distribution shifts. This advantage likely reflects the suitability of tree ensembles for structured clinical data: they capture nonlinear effects and feature interactions, tolerate outliers and missingness, and are relatively stable under routine hyperparameter tuning with moderate sample sizes. Under similar data settings, tree-based ensembles may therefore be preferable to deep networks as baseline models for clinical deployment.

This study used SHAP to provide a global interpretation of model predictions. Overall, the main contributors showed both shared and outcome-specific patterns, and several important predictors demonstrated generally consistent ranking patterns between the development and external validation cohorts. These findings support the stability and interpretability of the model across the two cohorts while also reflecting clinically meaningful feature associations with in-hospital adverse events. Across outcomes, inflammatory markers, particularly neutrophil and lymphocyte counts, were repeatedly ranked among the top predictors for AF, VF, and CA, consistent with evidence that systemic inflammation contributes to in-hospital instability after AMI [18,19]. There were significant differences across different tasks. These patterns align with established AMI biology, where inflammation reflects injury and repair, renal dysfunction and elevated natriuretic peptides reflect multi-organ stress and hemodynamic burden, and metabolic derangements are linked to adverse trajectories [20–22].

LCA provided a complementary, phenotype-based view of outcome heterogeneity by summarizing how adverse events co-occur at the subgroup level. The identified classes were clinically meaningful and aligned with prognosis [23]: phenotypes characterized by VF/CA were more closely linked to in-hospital death, whereas isolated AF tended to cluster in lower short-term risk profiles [24]. This is broadly consistent with common MACE-style framing, in which VF/CA and death represent higher-acuity events, while AF is often not included [25]. When interpreted alongside SHAP, these phenotypes also suggest mechanistic differences, with VF more related to infarct characteristics and patient factors, and CA/death reflecting downstream multi-organ stress and overall severity.

The models developed in this study offer a data-driven approach for early risk stratification of in-hospital adverse events in patients with AMI, with potential utility at both the bedside and system level. At admission or during early inpatient assessment, individualized risk scores can be generated from routinely available laboratory tests and vital signs to flag high-risk patients and support timely escalation of monitoring (e.g., intensified ECG surveillance, closer observation, or higher-acuity bed assignment), thereby reducing delayed recognition of serious events.

From a systems perspective, the model could be integrated into the Electronic Health Record as a clinical decision support module for automated risk calculation and visualization. This may facilitate rapid risk stratification and communication during routine care, and support resource allocation (e.g., ICU/CCU beds, telemetry capacity, and nursing intensity). In addition, individualized risk scores generated at admission may help clinicians prioritize patients requiring intensified ECG surveillance, closer bedside observation, or earlier escalation of care. For patients identified as high risk, the model may support earlier preventive interventions and more proactive monitoring strategies, whereas lower-risk individuals may continue with standard inpatient management. By providing rapid and explainable risk estimates based on routinely available structured clinical data, the framework may assist clinical workflow optimization and improve consistency in early inpatient risk assessment for AMI patients. With further validation and iterative updating, this framework may support more consistent, data-driven inpatient management for AMI.

This study has several limitations. First, the incidence of the four adverse events was low, resulting in marked class imbalance, particularly in the Xuanwu external cohort with limited positive cases. This may reduce model stability and hinder performance for rare outcomes. Although we used AUPRC-focused evaluation reliable prediction under very low event rates remains challenging. Second, the models relied mainly on structured variables and did not incorporate other informative data sources, such as imaging (e.g., coronary angiography, echocardiography), ECG signals, or clinical records. Integrating multimodal and longitudinal data may improve performance and broaden clinical applicability [26–28]. Third, external validation was conducted in a single center, which may not capture variability across regions, hospital types, practice patterns, and patient profiles. Further multicenter, international validation and prospective evaluation are needed to assess real-world effectiveness.

## Conclusions

In this retrospective study, we developed and externally validated explainable machine learning models to predict several in-hospital adverse events in patients with AMI. Using routinely collected clinical factors, tree-based models showed good discrimination across cohorts, and SHAP explanations helped identify key drivers of predicted risk. These models may support early risk stratification and inpatient resource planning for AMI, with clear potential for broad clinical implementation.

## Supporting information

S1 Fig DCA curves for predicting four in-hospital adverse events in the MIMIC-IV cohort. (A) AF. (B) VF. (C) CA. (D) In-hospital death.
(TIF)

S2 Fig Calibratio curves for predicting four in-hospital adverse events in the MIMIC-IV cohort. (A) AF. (B) VF. (C) CA. (D) In-hospital death.
(TIF)

S3 Fig SHAP dependence plots of the top six most important features identified in the best-performing model in the MIMIC-IV cohort. (A) Any in-hospital adverse event. (B–E) AF, VF, CA, and in-hospital death in the MIMIC-IV cohort, respectively.
(TIF)

S4 Fig ROC curves for predicting four in-hospital adverse events in the Xuanwu cohort. (A) AF. (B) VF. (C) CA. (D) In-hospital death.
(TIF)

S5 Fig DCA curves for predicting four in-hospital adverse events in the Xuanwu cohort. (A) AF. (B) VF. (C) CA. (D) In-hospital death.
(TIF)

S6 Fig Calibration curves for predicting four in-hospital adverse events in the Xuanwu cohort. (A) AF. (B) VF. (C) CA. (D) In-hospital death.
(TIF)

S7 Fig SHAP summary plots of the best-performing models identified in the MIMIC-IV cohort and applied to the Xuanwu cohort for external validation across different adverse outcomes. (A–D) AF, VF, CA, and in-hospital death in the Xuanwu cohort.
(TIFF)

S1 Table Demographic Characteristics of AMI Patients Stratified by the Occurrence of Four Adverse Events (Xuanwu Hospital Cohort) and Associated P-values. (XLSX)

S2 Table Final optimized hyperparameters and software environment for all models. (XLSX)

S3 Table Performance of RF, XGBoost, MLP, TabNet and LR for predicting four in-hospital adverse events (AF, VF, CA, and in-hospital death) in the MIMIC-IV cohort. (XLSX)

S4 Table Performance of RF, XGBoost, MLP, TabNet and LR for predicting four in-hospital adverse events (AF, VF, CA, and in-hospital death) in the Xuanwu cohort. (XLSX)
